# Virtual embodiment in fibromyalgia

**DOI:** 10.1038/s41598-023-36861-3

**Published:** 2023-07-03

**Authors:** Justyna Świdrak, Ana Arias, Edgar Rodriguez de la Calle, Antonio Collado Cruz, Maria V. Sanchez-Vives

**Affiliations:** 1 Fundació de Recerca Clínic Barcelona-Institut D’investigacions Biomèdiques August Pi I Sunyer, Barcelona, Spain; 2 Institute of Psychology Polish Academy of Sciences, Warsaw, Poland; 3 Rheumatology Service, Hospital Clínic de Barcelona, Barcelona, Spain; 4 ICREA, Barcelona, Spain

**Keywords:** Perception, Psychology, Fibromyalgia

## Abstract

Chronic pain alters the experience of owning a body and leads to disturbances in bodily perception. We tested whether women with fibromyalgia (FM) are receptive to bodily illusions of owning a visible and progressively invisible body in immersive virtual reality (VR), and what modulates this experience. Twenty patients participated in two experimental sessions; each session included two conditions in a counterbalanced order. We found that patients with FM could indeed experience virtual embodiment. Sentiment analysis revealed significantly more positive reactions to the progressively invisible body, yet twice as many patients declared they preferred the illusion of a visible virtual body. A linear mixed model revealed that the strength of embodiment was positively associated with body perception disturbances and negatively associated with FM symptoms intensity. No effect of pain during the VR experience nor interoception awareness on embodiment was found. The results indicated that patients with FM are receptive to virtual bodily illusions and that the impact of the embodiment depends on affective reactions, the level of cognitive body distortions, and the intensity of symptoms. Importantly, there is a large variation among patients which should be considered in future VR-based interventions.

## Introduction

Pain is a bodily sensation that connects the neural correlates of pain perception and body representation. It is not only a distinct sensation but also a piece of information about our bodies^1^. In fibromyalgia (FM), the most common symptoms are a generalized chronic pain in muscles and fibrous connective tissue (ligaments and tendons) described as ‘pain of everything’, joint stiffness, and chronic fatigue. Patients tend to report multiple soft tissue ‘tender points’ that are especially painful^2^. While specific pain location might change from 1 day to another, pain and fatigue are felt over the whole body. Patients with FM have enhanced sensitivity to aversive and non-aversive stimuli which is linked to altered interoception^3^. Cognitive modulation of pain and interoceptive perception can be influenced by various factors including attention, beliefs, conditioning, expectations, mood, and the regulation of the emotional response to sensory events^4–6^.

The perception of pain, its multisensory influences, non-aversive sensations arising from within the body, and the sense of body ownership are all closely related^7,8^. Thus, the intensity of pain can, to a certain degree, be modulated through bodily sensations and their manipulation. For example, in patients with chronic low back pain, visual feedback of one’s low back reduces pain intensity compared with visual hand feedback or controls^9^. Chronic pain is associated with changes in sensorimotor processing, mostly evident in the form of motor deficits, sensory changes, and body representation distortions^10,11^. Brun et al.^12^ demonstrated a positive correlation between pain intensity and conflict-induced sensorimotor disturbances, with patients with FM and complex regional pain syndrome (CRPS) being more prone to report disturbances than patients with arthritis and healthy participants. Patients with FM often present with an unstable body schema (the representation of a body for action planning and control)^13^, negative attitudes toward their bodies, and increased vigilance to their bodily signals^14^. Altogether, the body-pain relationship in patients with FM is complicated and can be described as a paradox:

[T]he intensification of fibromyalgia pain does in fact affect different aspects of body awareness: in particular, experienced body size, weight, and localization, as well as the experience of owning one’s own body. In addition, these disruptions in patient’s body awareness have as a result, a modification of the experience of pain, leading to the apparently paradoxical experience of being in pain while not feeling it^5^.

The sensation of ownership of our (real) body is, under physiological conditions, a stable experience that is critical for sensorimotor interactions with the environment. Despite such stability, it can be easily modified^15^, for example in the well-known rubber hand illusion, in which synchronous visuotactile stimulation leads to an illusion of ownership over a rubber arm^16^. Immersive VR has opened up further possibilities for inducing and experiencing body illusions^17^. In immersive VR technology, a computer-generated 3D audiovisual environment is experienced on a head-mounted display (HMD), replacing the user’s sensory inputs from the real world. In VR, a life-sized virtual body can be programmed to substitute the participant’s real body. Hence, when looking down towards themselves they will see a virtual body rather than their real one. Through real-time tracking the virtual body can be programmed to move synchronously and in correspondence with real body movements, and when an object is seen to touch the virtual body this touch can be felt on the real body (e.g. through vibrotactile stimulation). This setup typically gives rise to a sensation of ownership over the virtual body (termed embodiment). Embodiment consists of three subcomponents: the body ownership illusion (*This is my body*), agency (*I control my body*), and the sense of self-location (*I am where my body is*)^18^. It is still possible to experience an embodiment illusion when some of these conditions are modified, for example, when the user’s point of view is outside the body (e.g. in a third-person perspective)^19^ or the virtual body does not resemble one’s real body (e.g. in size, colour, or gender)^20^. The feeling of ownership over an external dummy or virtual body (or body part) has both physiological and behavioural consequences. For example, the vision of one’s own body can modulate pain perception^21^. Furthermore, seeing a virtual arm can significantly increase the thermal pain threshold, independently of attentional and stimulus adaptation processes^22,23^. When a subject observes the skin colour of their virtual arm dynamically changing, this can modulate pain sensitivity. In particular, a reddened arm significantly decreases the pain threshold compared with normal and bluish skin^23^.

A key feature of VR is the possibility of inducing manipulations that are otherwise impossible, such as making the body (semi)transparent, what can modulate the threshold to pain. In a study on healthy participants, Martini et al.^24^ demonstrated that viewing one’s semi-transparent body may reduce the ownership over that body. Still, participants that did report stronger body ownership of a semi-transparent body had a lower pain threshold in this condition, an observation that was suggested to be related to the unpredictability of potential harmful stimuli.

If owning a semi-transparent virtual body increased pain sensitivity (or decreased pain threshold), this would not seem a good strategy to treat chronic pain. However, the modulation of pain threshold that was observed in healthy subjects is not a good predictor of how such manipulations influence chronic pain. For example, making a virtual arm semi-transparent diminished pain ratings in patients with CRPS, but had no effect in patients with peripheral nerve injury^25^. This suggests that body transparency may modulate pain under certain conditions, and such manipulations are increasingly being tested in pain research and treatment^26–28^.

Some fibromyalgia patients report a disruption in their body perception, leading to *disembodiment*^5^, as a result of the body in FM being painful and ‘problematic’. Here, our aim was to evaluate whether it is possible for patients with FM to experience being *embodied* in a virtual body and then *disembodied* by observing it progressively becoming invisible. In a second step, we investigated this potential dual role of progressive invisibility in lowering pain threshold (as demonstrated in the pain-free population) while decreasing pain intensity (as demonstrated in patients with CRPS). Since the population of patients with FM is characterized by high heterogeneity of both psychological and physiological characteristics^29^, we applied a mixed methods design which allowed for a deeper understanding of the unique experience.

## Methods

The study was carried out in compliance with the Declaration of Helsinki (Fortaleza, Brazil, October 2013) in addition to the Law 14/2007 of July 3 on Biomedical Research. The experimental protocol was approved by the Clinical Research Ethics Committee of the Hospital Clínic of Barcelona (HCB/2021/0501). Written informed consent was obtained from all patients before being included in the study.

We carried out a within-group experiment with two conditions, the visible and progressively invisible bodies, presented in a counterbalanced order across two sessions. Repeated measures were used to control for the inference of the novelty effect of VR, the order of conditions, and daily fluctuations of symptoms.

### Sample

Twenty-one female patients with FM were recruited for the study. The sample size was established on the basis of the minimal sample size (*N* = 15) calculated in g*Power 3.1 for a repeated-measures ANOVA (within factors), assuming effect size *d* = 0.4 and power 0.80.

The inclusion and exclusion criteria can be found in Supplementary Information (p.1). One patient withdrew from the study, due to the physical discomfort experienced in the first session, and thus 20 patients were included in the analyses. The mean age of the group was 43 (SD = 5) years (min = 36, max = 55), and the mean number of years lived with the diagnosis of FM was 9 (SD = 12). Two patients claiming that they had symptoms all their life, but the majority (12 patients) were diagnosed within the last 5 years. The group had diverse levels of education, from primary school (*N* = 5) to secondary or high school (*N* = 8) to a university degree (*N* = 5; other *N* = 1).

### Study design and experimental procedure

**Figure 1:**
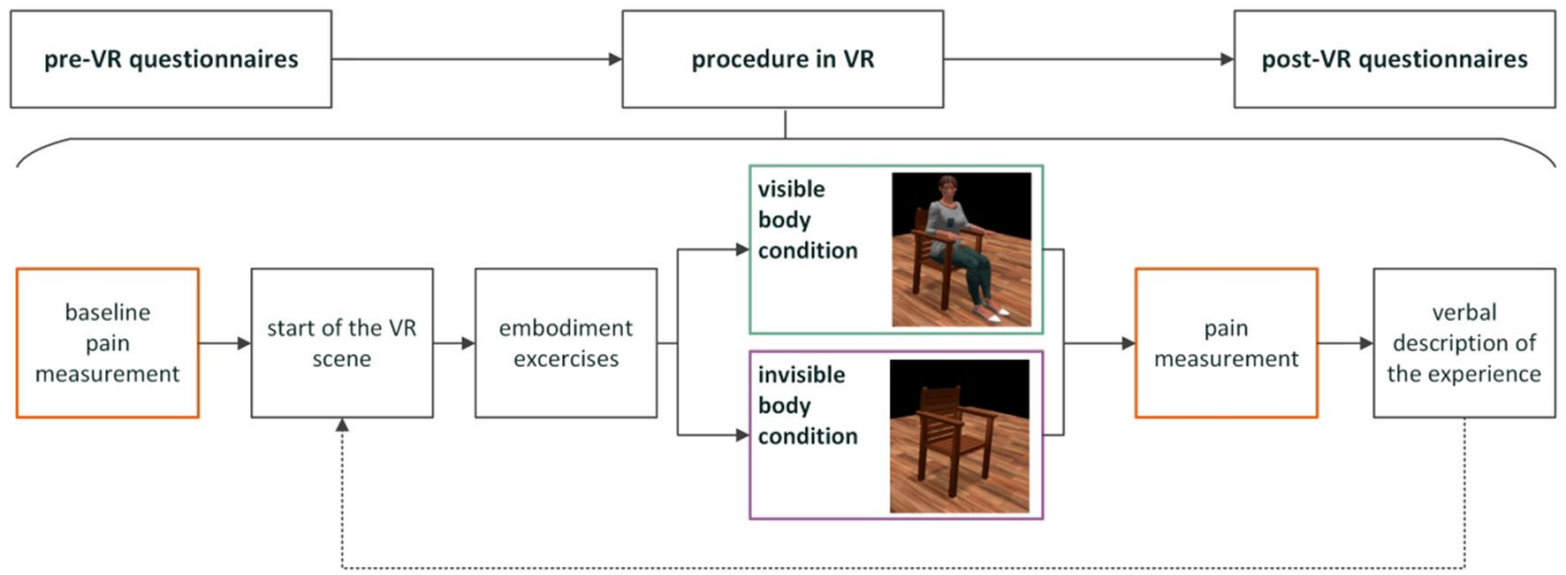
Experimental procedure.

The experiment consisted of two identical sessions that lasted approximately 30 min each. There was 1 week between the sessions. The study was carried out at the Hospital Clinic Barcelona, and a COVID-19 protocol was applied to minimise cross-infection risk. Data were collected from April to September 2021. Participants were greeted by the experimenter and asked to read and sign the patient information sheet and the informed consent form. The demographic data were collected separately from the consent form. Patients then filled in the pre-study questionnaires and were instructed how to use the head-mounted display (HMD). Finally, the VR scene was displayed (Fig. 1).

**Figure 2:**
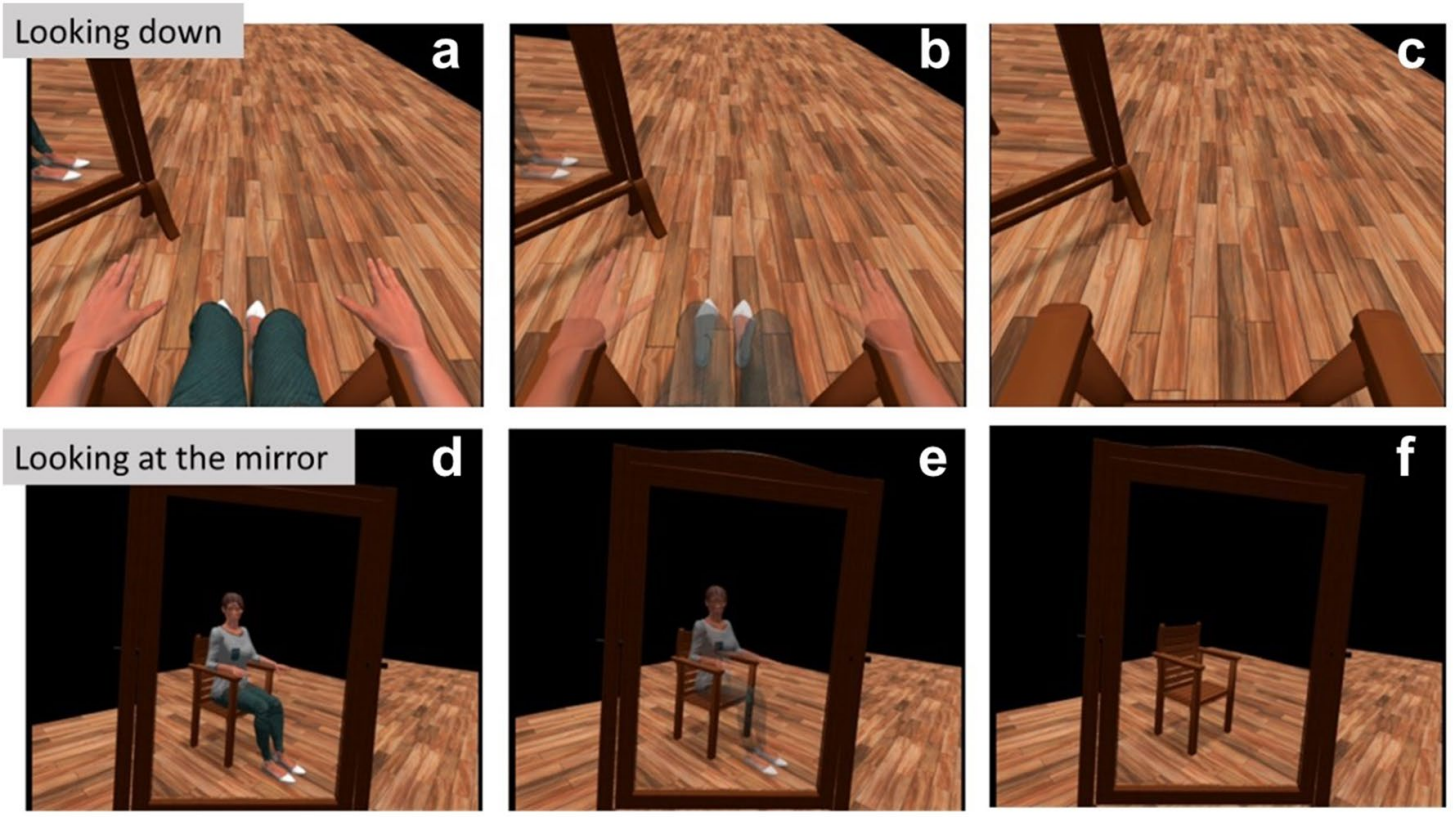
Virtual reality scene. (**a–c**) Looking down at one’s body. (**d–f**) Observing one’s body in the mirror. (**a,d**) control condition; (**b**,**e**) gradual increase in transparency, (**c**,**f**) the body becomes fully transparent.

There were two scenes, one with a *visible body* and one with the progressively invisible body condition (called *invisible body*) presented in a pseudo-randomized order (Fig. 2). During the experience, participants were asked three times about their current pain level (immediately after donning the HMD and after each scene). After each condition, they were also asked to respond out loud and describe how they felt during the experience. Their answers were recorded in an audio format. After the last pain measurement, the experience ended, and the experimenter assisted participants with the removal of the equipment. Finally, participants answered several embodiment-related questions and indicated their preferred scene. Finally, they were debriefed and thanked for their participation.

### Procedure in virtual reality

1. Participants saw a calibration screen (10 s).
2. Participants were asked about their pain level (baseline) and responded by selecting a number from 0 to 100 on a visual analogue scale (15 s).
3. The virtual room scene was displayed, and participants could see their virtual body sitting on the chair from the first-person perspective that moved in synchrony with their real body (15 s).
4. Participants were asked to do simple movements (30 s) (“look at your right hand, look at your left hand, look at yourself in the mirror, stretch your right arm, stretch your left arm”).
5. Four cubes appeared in front of the participant, who was asked to touch them with their virtual hands. When touched, the object disappeared, the corresponding controller vibrated, and a short sound was played.
6. Then, depending on the group and the condition, participants observed one of the following:
  - their normal virtual body (*visible body condition*) (100 s);
  - how their body gradually becomes transparent (*invisible body condition*) (30 s of gradually increasing transparency + 70 s 100% transparency);
7. Participants were asked about their level of pain.
8. Participants were asked an open question to explain their sensations and thoughts during the scene.
9. The second scene loaded in a pseudo-randomized order. After the scene, the experience ended.

### Measurements

#### Measures before the VR experience (pre-study)

The Fibromyalgia Impact Questionnaire (FIQ) is a questionnaire developed to estimate the current health status of women with FM syndrome in clinical and research settings. The FIQ has been translated and validated into 14 different languages and has been used in hundreds of research articles as an outcome measure. A Spanish version of the latest, revised FIQ was validated in 2013 by Salgueiro et al.^30^ It consists of 21 questions that measure functioning, overall impact, and symptoms. In this study, to keep the procedure as short as possible, we used only part 3, which estimates the severity of the 10 most common symptoms in the last 7 days. Total scores range from 0 to 100 points.

Interoceptive awareness was measured with the Multidimensional Assessment of Interoceptive Awareness (MAIA) scale, a self-report questionnaire evaluating various aspects of interoceptive awareness. There are eight subscales: Noticing, Not Distracting, Not Worrying, Attention Regulation, Emotional Awareness, Self-Regulation, Body Listening and Trusting. We used the 30-item Spanish adaptation of the scale^31^. Individual scores range from 0 to 150.

Body perception distortions were measured with the Fremantle Back Awareness Questionnaire (FBAQ)^32,33^ in a slightly adapted version, where we replaced the word *back* with the word *body* (Supplementary Table S1)*.* The unidimensional scale measures distortions of body image, including perceived changes in size, swallowing, or lack of control over one’s movements. The minimal total score is 0 and the maximum is 36.

#### Measures during the VR experience

Pain was measured on a visual analogue scale ranging from 0 (no pain) to 100 (most intense pain imaginable)^34^:

- *Please indicate the intensity of pain you are feeling right now. A value of 0 means "no pain", and a value of 100 means "the worst pain imaginable". Select the desired value using the joystick on the right controller and confirm by pressing the trigger.*

During the VR experience**,** after each condition, participants responded out loud to one of the corresponding questions: *What did you feel when you had a virtual body?* and *What did you feel when your body became transparent and disappeared?* Answers to the open questions asked during the VR experience were recorded in audio form and then manually transcribed.

#### Measures after VR experience (post-study)

Some measures were taken afterwards to shorten the time spent in HMD and reduce the overall difficulty of the experimental procedure. The illusion of virtual body ownership was measured after the virtual reality experience on three Likert scale questions, using a scale from 1 (not at all), to 7 (very much):

- I felt that the virtual body I saw when I looked down was my own body (ownership).
- I felt that the movements of my virtual body were caused by my own movements (agency).
- To what extent were the sensations of your real body surprising and unexpected? (surprising reactions).

Open questions were asked to better understand the experience of patients and determine their preferred scenario.

- *Which of the two scenes did you prefer, which one did you like the most, and why?*

### Apparatus

The Oculus Quest headset uses an OLED panel with a resolution of 1600 × 1440 per eye display. It has a refresh rate of 72 Hz and weighs 470 g, 6 degrees of freedom, and rotational and positional tracking. To track hand movements, we used the Oculus Quest controllers, which are comfortable and easy to use. Oculus Quest does not need a computer to run the applications and has a comfortable in-ear speaker system, which makes use of the system user-friendly.

We used CleanBox CX1 to disinfect the system. It uses ultraviolet C light-emitting diodes to disinfect surfaces from viruses, bacteria, and fungi.

### Statistical analyses

We adapted a mixed methods approach to analyse the answers to the open questions transcribed verbatim by running a sentiment analysis (sentiment-analysis-spanish v0.0.25 python library) and clustering the content of the answers into nine main themes related to affective and physiological states. A repeated-measures ANOVA was used to test the differences in sentiment between session and conditions. Differences in symptoms, interoception awareness, body perception disturbances, and embodiment between sessions were verified with the Friedman test. A random intercepts and random slopes mixed linear model was built to estimate the effect of ongoing pain, symptom intensity, interoceptive awareness, and body perception disturbances on embodiment (python statsmodels library)^35^. The model assumptions were tested visually and formally with the Shapiro–Wilk test for normality and the White Lagrange multiplier test for heteroscedasticity.

## Results

### Sample characteristics and their influence on embodiment and pain

There were no differences between session 1 and 2 in any of the baseline questionnaire scores. Patients evaluated the intensity of their symptoms (FIQ-3) as medium to high (M = 6.09, SD = 1.30, and M = 6.02, SD = 1.85 in session 1 and 2 respectively) (Table 1). Moreover, the body perception disturbances were experienced by them rarely or occasionally, although the variance was large, with some patients reporting no disturbances and others scoring almost the maximal possible score (max score in session 1 = 3.78 and in session 2 = 1.87). The mean level of interoceptive awareness was slightly above the middle of the scale (session 1: M = 3.06, SD = 0.55, session 2: M = 2.99, SD = 0.61).

**Table 1:** Sample characteristics.

Scale	Min–max mean score	Session 1		Session 2		Friedman’s *W*
		M	SD	M	SD	
FIQ-3	0–10	6.09	1.30	6.02	1.85	0.129
FBAQ	0–4	1.81	0.90	1.67	0.90	0.074
MAIA	0–5	3.06	0.55	2.99	0.61	0.010

Min–max mean score		Med	IQR	Med	IQR	Friedman’s *W*
Body ownership	1–7	3	5	3	5	0.705
Agency	1–7	6	2	5.5	3.25	0.405
Surprising reactions	1–7	6	2.5	5	3.75	0.248

*M* mean, *SD* standard deviation, *Med* median, *IQR* interquartile range.

**Figure 3:**
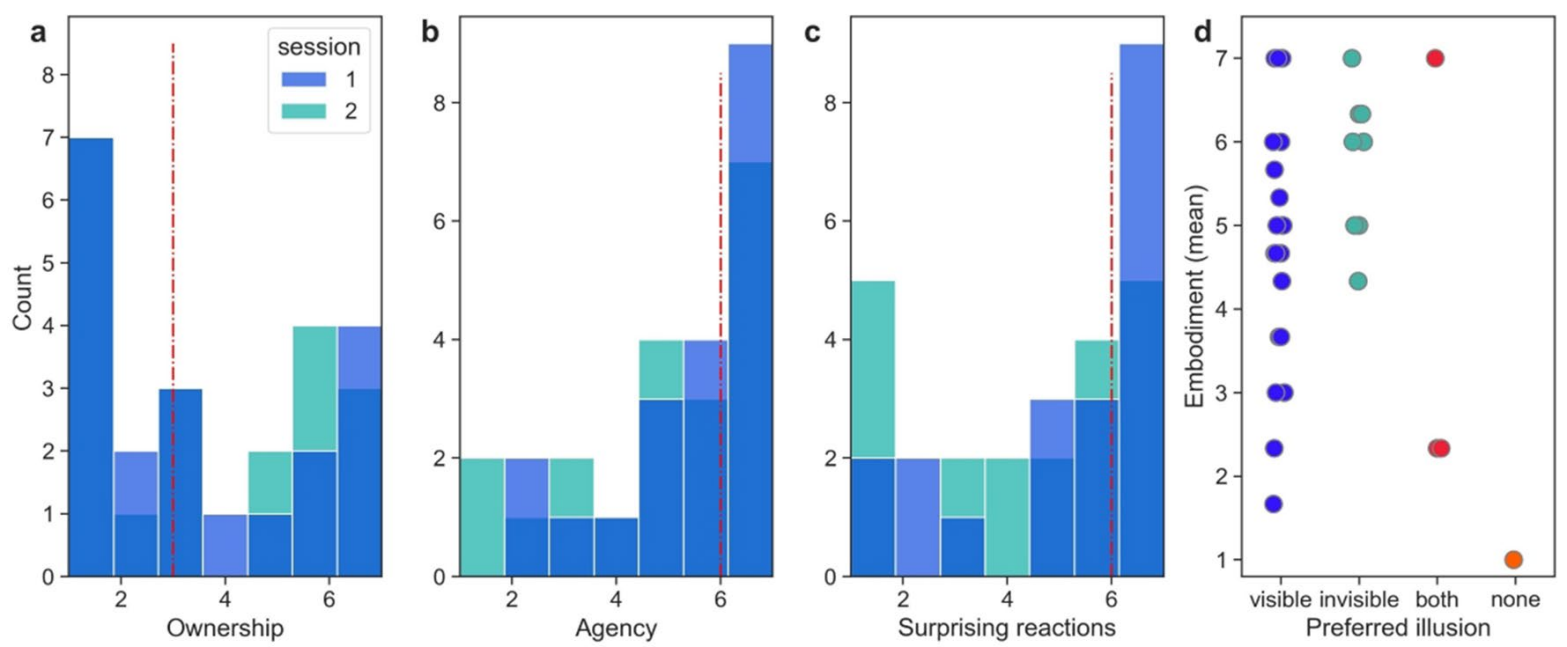
(**a**) Distribution of body ownership values on a Likert scale (1–7) in the sample. (**b**) Distribution of agency over the virtual body on a Likert scale (1–7). (**c**) Surprising responses on a Likert scale (1–7). In (**a–c**) the first and second sessions are represented. The red line corresponds to the median. (**d**) Embodiment levels and the preference for bodily illusion.

Embodiment was scored on a Likert (ordinal) scale and the data points were not normally distributed (*p* < 0.05), thus we applied non-parametric tests. In any case non-parametric tests are more conservative than parametric, in the sense that null hypothesis rejected by a parametric test might not be rejected by the equivalent appropriate non-parametric test. In both sessions, patients rated their overall embodiment (Fig. 3d) as medium to high (session 1: M = 4.78, SD = 1.67, session 2: M = 4.37, SD = 1.93) (Table 1). Looking at the answers to each embodiment question, we found that the lowest scores and the largest variance were observed for ownership (Fig. 3a; median = 3, IQR = 5) with a large group of participants scoring either very low (< 2) or very high (> 5), while agency (Fig. 3b) and vivid reactions (surprising, unexpected; Fig. 3c) were rated mostly high, especially in session 1, perhaps due to the fact that participants were unfamiliar with the scenarios and because they had actual agency over their virtual body, since their virtual body moved with them.

Half of the participants in sessions 1 and 2 preferred the experience of the visible body experience (*N* = 11 and *N* = 10, respectively). This group reported a wide range of embodiment scores (min. 1, max 7), with a median of 4.3 (IQR = 3) in session 1 and 5 (IQR = 1.3) in session 2. The 25% of the group who chose the scene where their body became progressively invisible reported a high level of embodiment (session 1: median = 6, IQR = 1.3; session 2; median = 6.2, IQR = 0.6). Some patients did not respond (*N* = 3) or preferred both or none of the sessions equally (Fig. 3).

### Content analysis

**Figure 4:**
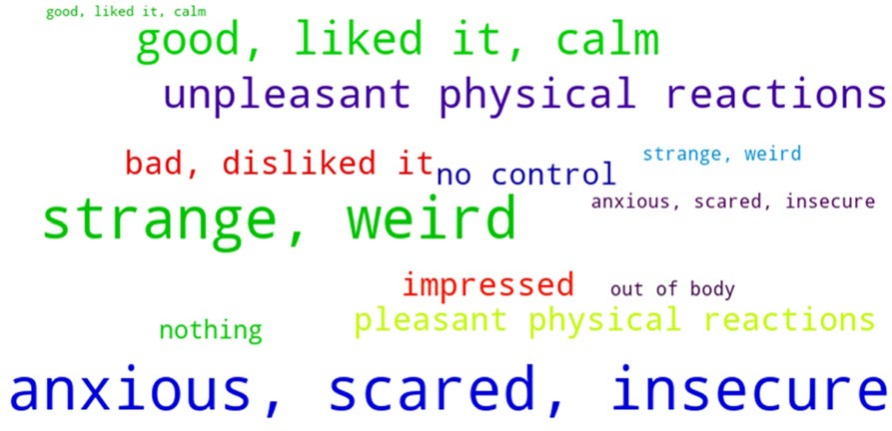
Word cloud using frequency of words and expressions used to describe the experience in VR. A word which appears twice is 50% bigger (scale = 0.5).

We analysed the content of the answers to the open questions by selecting words and short phrases which described affective reactions, physiological states, or were related to bodily experiences. Next, we grouped them into nine general categories and counted the frequencies of their content (Fig. 4).

Overall, responses were very diverse and stretched from very negative (e.g. horror, anguish, fear, tension) to neutral (strange, different) to very positive (relief, calm, light, good feeling). Looking at the complete answers, we noticed an important role for the meaning or interpretations patients gave to the virtual scenarios. For example, a few patients associated their disappearance with death, which resulted in overall negative affective reactions. Others, who had a more neutral or no interpretation, reacted in a neutral or even positive manner. Many patients had various physical reactions, from dizziness to a weightlessness, from increased pain to no pain. Four patients said they felt nothing and four did not respond at all (Table 2).

**Table 2:** Classification of words and phrases related to affective reactions, physiological states, or bodily experiences.

Category	Word/expression (original in Spanish)	Frequency	Total
Bad, disliked it	I don’t like it (no me gusta)	3	7
	Unpleasant (mal rollo)	1	
	Displeasing (desagradable)	2	
	Discomfort (malestar)	1	
Unpleasant physical reactions	Rigid (rígida)	2	12
	Tension (tensión)	3	
	Dizziness (mareo)	2	
	Disgust (asco)	1	
	Pain (dolor)	1	
	More sore (más dolorida)	1	
	Emptiness (vacío)	1	
	Vertigo (vértigo)	1	
Anxious, scared, insecure	Anxiety (ansiedad)	11	26
	Anguish (agustia)	7	
	Fear (miedo)	4	
	Associated with death (asociado con muerte)	2	
	Stressed (estresada)	1	
	Horror (horror)	1	
No control	Overwhelm (agobio)	3	6
	Out of control (sin control)	3	
Rare, strange, weird	Rare/weirdness (rara/rareza)	12	25
	Strange (extraño)	8	
	Uncomfortable (incómoda)	2	
	Uncertainty (incertidumbre)	1	
	Insecurity (inseguridad)	1	
	Nervousness (nerviosismo)	1	
Nothing	Nothing (nada)	4	4
Out of body	I’m not inside (no estoy dentro)	1	2
	Out of body (fuera de cuerpo)	1	
Impressed	Impressed (impresionada)	3	7
	Astonishment (asombro)	1	
	Different (diferente)	1	
	Surprised (sorprendida)	2	
Good, calm, liked it	Calm (tranquila)	4	14
	Good feeling (buena sensación)	3	
	Relief (alivio)	2	
	Relaxed (relajada)	2	
	Calm (calma)	1	
	Much, much better (muchísimo mejor)	1	
	Very good (muy bien)	1	
Pleasant physical reactions	Float (flotar)	1	7
	Light (ligera)	2	
	Weightlessness (ingravidez)	1	
	Focus on body (centrarme en cuerpo)	1	
	More strength (más fuerza)	1	
	No pain (sin dolor)	1	

### Sentiment analysis

**Figure 5:**
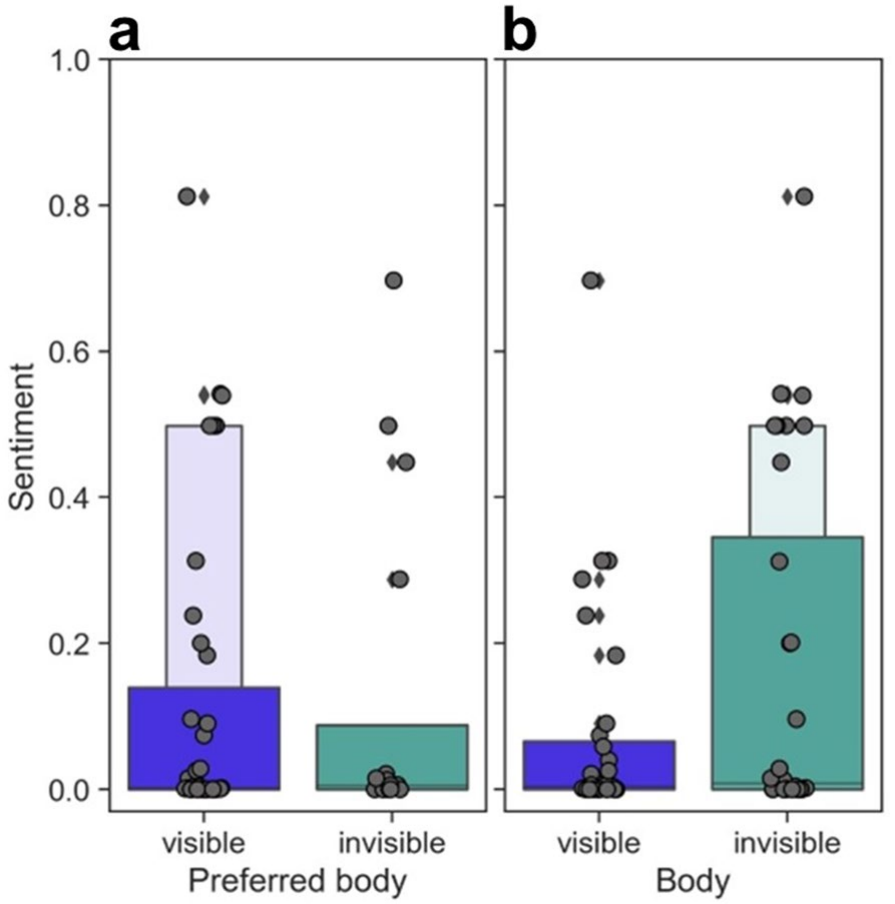
Box plots representing (**a**) the distribution of sentiment scores by preferred body illusion and, (**b**) the body illusion participants referred to.

In the second step, we calculated the sentiment scores in the answers to open questions for each condition on a scale from 0 (very negative) to 1 (very positive). The mean sentiment was rather negative (mean = 0.11, SD = 0.19). Furthermore, repeated measures ANOVA yielded significant differences between bodily illusions (*F*(1,15) = 5.21, *p* = 0.037, eta squared = 0.258), with the invisible body evoking more positive reactions (mean = 0.14, SD = 0.23) than the visible one (mean = 0.07, SD = 0.14). There were no differences between sessions. However, the most positive sentiment was recorded among patients who indicated that visible body conditions were preferred (Fig. 5).

### Factors modulating the embodiment illusion

To test the influence of measured variables on embodiment, we built a mixed effects model using the mixedlm in python statsmodels library, following Harrison et al.^36^. In the first step, we standardized the questionnaire scores (FIQ-3, FBAQ, MAIA) so that they had M = 0 and SD = 1^37^. Next, we fitted a random intercept and random slope model for session, with observations clustered in individuals and fixed (session) and random (mean pain during VR, fiq_z, fbaq_z, maia_z) factors. The model formula included the embodiment as a dependent variable, and the mean pain in VR, FBAQ, FIQ, MAIA, and session as factors. (*model* = *"emb_z* ~ *pain_mz* + *fbaq_z* + *fiq_z* + *maia_z* + *C(session)", re_formula* = *"C(session)").*

#### Normality of the residuals and heteroscedasticity check

Both visual analysis and formal statistical tests confirmed that the assumptions were not violated. In particular, the Shapiro–Wilk test for normality of the residuals (*W* = 0.952, *p* = 0.087) and the White’s Lagrange multiplier test for heteroscedasticity (LM = 25.95, *p* = 0.132, *F* = 1.94, *p* = 0.074) were not significant.

#### Model parameters

We calculated the intraclass correlation coefficient (ICC), which measures the similarity of the responses within a random effect (Table 3). In our model, the ICC for the session is equal to 0.490, suggesting a moderate correlation. The random effect of the participant was 0.600, and the embodiment varied on average 0.775, which indicated a large variation between and within participants. The estimated correlation coefficient between the random intercepts and random slopes was − 0.658 which indicated that patients experiencing high embodiment in the first session experienced lower embodiment in the second session. Following Westfall et al. (2014), we calculated the effect size for a design with random participants and random items *d* = 0.34, which is small.

**Table 3:** Mixed linear model.

	Coef	Std. err	*z*	*p* >*|z|*	[0.025	0.975]	SD
Intercept	0.076	0.224	0.338	0.735	− 0.363	0.514	0.438
C(session) [T.2]	− 0.152	0.225	− 0.672	0.501	− 0.593	0.290	0.442
pain_mz	0.262	0.171	1.534	0.125	− 0.073	0.597	0.335
fbaq_z	0.585	0.229	2.555	0.011	0.136	1.034	0.449
fiq_z	− 0.461	0.212	− 2.171	0.030	− 0.876	− 0.045	0.416
maia_z	0.084	0.208	0.404	0.686	− 0.323	0.491	0.407
id Var	0.600						
id x C(session) [T.2] Cov	− 0.224						
C(session) [T.2] Var	0.193						

Pain_mz, mean pain during VR (standardized); fbaq_z, Fremantle Back Awareness Questionnaire (adapted) (standardized); fiq_z, Fibromyalgia Impact Questionnaire (part 3) (standardized); maia_z, Multidimensional Assessment of Interoceptive Awareness (standardized); id, participant’s id.

**Figure 6:**
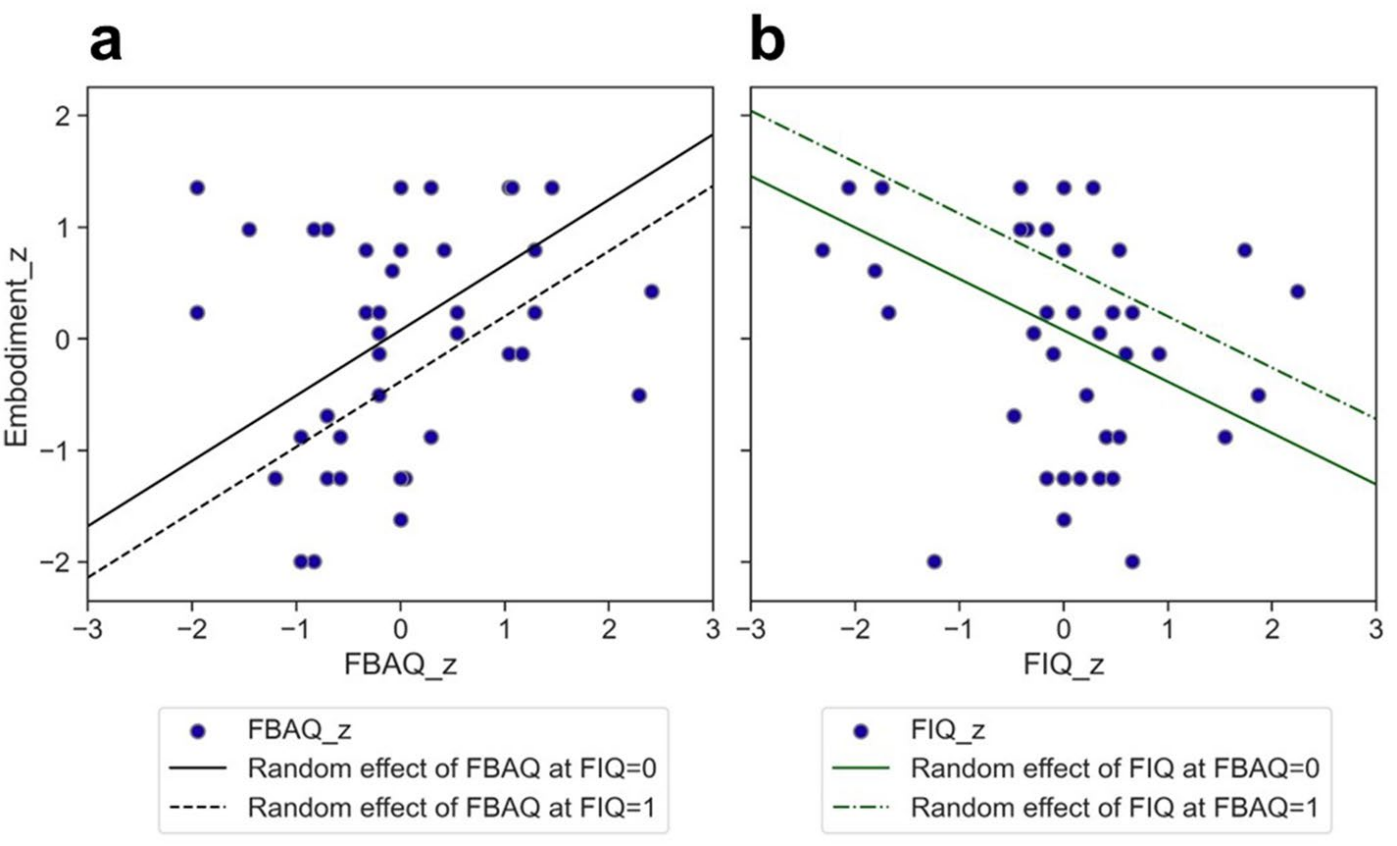
Random effect of body perception disturbance (**a**) and FM symptoms (**b**) on embodiment.

We found significant random effects of fbaq_z and fiq_z, indicating that more body perception disturbances led to higher embodiment levels, while more intensive FM symptoms (reported for the last 7 days) had the opposite effect (Fig. 6). Neither pain nor interoception awareness were significant.

To obtain the explained variance, we followed the approach proposed by Nakagawa and Schielzeth^38^ and calculated the marginal and conditional R^2^_GLMM_, which allows separation of the contributions of fixed and random effects. In our model, the marginal *R*^2^_GLMM_ = 18.86% of the variance explained by fixed factors and the conditional *R*^2^_GLMM_ = 67.84%, which can be interpreted as the variance explained by the entire model (both fixed and random factors).

## Discussion

FM is an intriguing and still rather poorly understood chronic pain condition. In this study, we explored the experience of owning a body and observing it become progressively invisible in women with FM. We found that patients with FM can experience embodiment illusions in immersive VR in both visible and invisible conditions, and in spite of their chronic pain. We also observed that these experiences led to diverse reactions on both affective and physiological levels and that cognitions, and perhaps also metacognitions^39^, may be involved in modulation of these reactions. Many patients described their state as anxious or strange, while others felt relaxed. A wide range of bodily sensations was also mentioned, including changes in weight, such as a feeling of weightlessness or floating, in contrast to the commonly experienced feeling of heaviness or swelling^40^. Although the progressively invisible body illusion evoked more positive sentiment, it was not reflected in behaviour, operationalized here as the preference of one of the illusions, as patients more frequently chose the visible body condition.

Fuchs and Schlimme^41^ distinguished two forms of disturbed body awareness in the context of schizophrenia, namely disembodiment and hyper-embodiment. The first is experienced as a disconnection with one’s body (alienation) and the latter refers to the experience of being totally immersed in it, a feeling of the physiological experiences being “too real”, which can be both pleasant and painful, depending on the context. Such hyper-embodiment is a daily experience of patients with FM, as their bodies are often felt to be an unavoidable obstacle, rigid and heavy^42^. In our study, the progressive invisibility triggered in several participants a feeling of weightlessness and relief. We speculate that in these patients the constant experience of hyper-embodiment was broken or transformed into a pleasant feeling of *virtual disembodiment* (“no body” ownership). Nonetheless, for other participants the experience was rather unpleasant and attracted their attention towards their symptoms, perhaps due to the multisensory conflict caused by the absence of visual cues (invisible body) during ongoing proprioceptive and painful stimulation.

The most important result was that higher levels of body perception disturbance predicted a higher embodiment. While the cause of this is not known, we can speculate. Valenzuela-Moguillansky^5^ hypothesized that disruption of implicit knowledge of the topography of patients’ bodies leads to a paradoxical feeling of being in pain while not feeling it. A disturbed body image is a signal of a desynchronization of brain and body, which is one of the main characteristics of FM^43^. We speculate that body distortions are a form of plasticity in body representation, and thus some patients with FM may be more prone to incorporate virtual bodies, which is also a form of plasticity^17,44^. Importantly, FM not only affects interoception, but also exteroception, and reduces the tendency to actively listen to the body for insight^45^. We suggest that disturbances in body awareness might lead to increased sensitivity to external cues such as strong visual stimulation in VR. However, more studies are required to validate this preliminary result.

Interestingly, while stronger fibromyalgia symptoms (including pain) in the prior week decreased embodiment, the pain ratings reported during the virtual experience had no effect on the embodiment. This suggests that ongoing musculoskeletal pain can be partially overridden by strong synchronous visuomotor and visuotactile stimulation, confirming that chronic pain does not completely prevent embodiment^27^. However, whether very intense or acute pain prevents virtual embodiment, deserves further investigation.

We observed a large variation between participants and sessions. The patients who reported higher embodiment in the first session rated the strength of the illusion as lower in the second session. We speculate that in the first session, some participants were surprised or baffled with the illusion, but the novelty effect had worn off in the second exposure.

An important contribution of this study is a demonstration of usefulness of the mixed methods in chronic pain research, obtained here by combining sentiment analysis with a quantitative and qualitative text analysis and multilevel modelling. The collected data helped us to improve our understanding of the bodily experiences among patients with FM and added context to the large variability of variance observed in the questionnaires.

The study had several limitations. We chose to keep the duration of the procedure as short as possible to minimize fatigue and discomfort of the patients^28^. For the same reasons, we only measured overall pain, without specifying the location or type of pain. Future studies dedicated to embodiment and pain modulation should apply a standardized scale, such as the one proposed by Gonzalez-Franco & Peck^46^ and the short form of the Brief Pain Inventory^47^. We also take note of the weak effects obtained in the mixed linear model. Although the sample was sufficient to obtain a converged model with satisfactory parameters, a larger sample is recommended in future studies.

Four patients reported that they did not feel embodied because the virtual avatar did not look like them. Although the embodiment illusion is normally triggered automatically at a low level if the basic conditions are accomplished (namely a first-person perspective, human-shaped body, congruent visuomotor/visuotactile cues^48^), a greater similarity of the avatar to the subject may be important in the case of chronic pain patients who are less sensitive to virtual embodiment. Thus, future studies should investigate the role of look-alike avatars in embodiment among patients with FM.

Future studies should also investigate the sources of variability among patients with FM in the context of body perception and embodiment to identify potential subgroups of patients who may be more sensitive to specific types of virtual bodily illusion. Although several subtypes of patients have already been identified^29^, it is unclear whether these profiles can explain the differences observed in experiencing virtual embodiment. Another interesting research path which has not received much attention until now is the role of cognition and metacognition in embodiment among both clinical and healthy populations, especially in scenarios which offer limited context and broad space for interpretation. Moreover, in future more attention should be paid to the rich affective reactions patients present during the virtual experience. In general, virtual embodiment is a promising tool for diagnosing and treating some of the least understood symptoms of FM related to the bodily self and its disturbances which have a severe impact on patients’ quality of life.

## Conclusions

Women with fibromyalgia are characterized by a large intra- and interpersonal variety which leads to diverse affective reactions to the same bodily illusion in virtual reality. Fibromyalgia symptoms intensity may negatively affect the proneness to virtual embodiment. Paradoxically, more disturbed body perception may strengthen the embodiment illusion. Moreover, pain reported during the virtual experience as well as interoception awareness did not affect the strength of virtual body illusions.

## Supplementary Information

Supplementary Information.

## Data Availability

Data are available on request from the corresponding author.
