# Supplementary material for: Virtual embodiment in fibromyalgia
Source: Sci Rep. 2023 Jul 3;13:10719. doi: 10.1038/s41598-023-36861-3 (PMC10318080; doi:10.1038/s41598-023-36861-3)
Supplement: Supplementary file 1 — Supplementary Information. [file 41598_2023_36861_MOESM1_ESM.docx]

**Virtual embodiment in fibromyalgia**

**Supplementary information.**

Supplementary Methods

Inclusion criteria:

- Age between 35 and 60 years of age.
- Diagnosed with fibromyalgia in the Hospital Clinic (Unidad de Sensibilización Central-Fibromialgia) according to the2010 criteria of the American College of Rheumatology (Wolfe et al., 2010):
  - widespread pain, and
  - some of the following: fatigue, waking unrefreshed, cognitive symptoms (e.g. memory, verbal fluency, concentration problems).
- Symptoms have been present at a similar level for at least 3 months.
- The patient does not have a disorder that would otherwise explain the pain.
- The patient visits the hospital because of her ongoing treatment (is not classified as the high-risk COVID-19 group).

Exclusion criteria:

- pain of other origin (e.g., rheumatoid arthritis; post-surgery pain),
- current or prior cerebral disease (e.g., stroke, cerebral haemorrhage, head trauma, epilepsy),
- frequent dizziness and/or headaches,
- severe psychological disorders (including severe depression, fear of heights),
- driving a car/motorbike/bicycle directly after the VR experience,
- working with dangerous or complex machinery directly after the VR experience,
- pregnant patients,
- ongoing COVID-19 infection,
- current severe flareup of pain levels.

Supplementary Table S1. English and Spanish adaptation of the Fremantle Back Awareness Questionnaire to the whole-body perception disturbances.

| English original | English adapted for the whole body | Spanish translation of the adaptation |
| --- | --- | --- |
| 1. My back feels as though it is not part of the rest of my body. | 1. My body feels as though it was not part of myself. | 1. Mi cuerpo se siente como si no fuera parte de mí. |
| 2. I need to focus all my attention on my back to make it move the way I want it to. | 2. I need to focus all my attention on my body to make it move the way I want it to. | 2. Necesito centrar toda mi atención en mi cuerpo para que se mueva como yo quiero. |
| 3. I feel as if my back sometimes moves involuntarily, without my control. | 3. I feel as if my body sometimes moves involuntarily, without my control. | 3. Siento como si mi cuerpo a veces se moviera involuntariamente, sin mi control. |
| 4. When performing everyday tasks, I don't know how much my back is moving. | 4. When performing everyday tasks, I don’t know how my body is moving. | 4. Al realizar las tareas cotidianas, no sé cómo se mueve mi cuerpo. |
| 5. When performing everyday tasks, I am not sure exactly what position my back is in. | 5. When performing everyday tasks, I am not sure exactly of my body’s position. | 5. Al realizar las tareas diarias, no estoy seguro de la posición exacta de mi cuerpo. |
| 6. I can't perceive the exact outline of my back. | 6. I can’t perceive the exact outline of my body. | 6. No puedo percibir el contorno exacto de mi cuerpo. |
| 7. My back feels like it is enlarged (swollen). | 7. Some parts of my body feel like they are enlarged (swollen). | 7. Algunas partes de mi cuerpo se sienten como si estuvieran agrandadas (hinchadas). |
| 8. My back feels like it has shrunk. | 8. My body feels like it has shrunk. | 8. Mi cuerpo se siente como si se hubiera encogido. |
| 9. My back feels lopsided (asymmetrical). | 9. My body feels lopsided (asymmetrical). | 9. Mi cuerpo se siente desbalanceado (asimétrico). |
| 0 = Never  1 = Rarely  2 = Occasionally  3 = Often  4 = Always | 0 = Never  1 = Rarely  2 = Occasionally  3 = Often  4 = Always | A continuación, se muestran distintas impresiones dadas por pacientes acerca de cómo perciben su cuerpo. Utilizando la siguiente escala, por favor indica hasta qué punto siente su cuerpo de la manera descrita cuando experimenta dolor en él.  0 = Nunca se siente así  1 = Raramente se siente así  2 = Ocasionalmente, o parte del tiempo se siente así  3 = A menudo, o una cantidad moderada de tiempo se siente así  4 = Siempre, o la mayoría de las veces se siente así |
